# Earning the Trust of African American Communities to Increase Representation in Dementia Research

**DOI:** 10.1093/geroni/igaa057.3244

**Published:** 2020-12-16

**Authors:** Elena Portacolone, Nynikka Palmer, Peter Lichtenberg, Catherine Waters, Carl Hill, Sahru Keiser, Julene K Johnson

**Affiliations:** 1 University of California San Francisco, San Francisco, California, United States; 2 Wayne State University, Detroit, Michigan, United States; 3 Alzheimer’s Association, Chicago, Illinois, United States

## Abstract

Black/African American populations are underrepresented as participants in dementia research. A major barrier to participation of African American older adults in dementia research is a tendency to distrust research institutions owing to a legacy of racism. Building on the Ford framework, the objective of our study was to examine factors that influence participation in dementia research among African American older adults and caregivers, with an emphasis on understanding factors related to trust. Data were collected from 10 focus groups with African American older adults (n=91), 5 focus groups with caregivers (n=44), and interviews with administrators of community-based organizations (n=11), and meetings with our Community Advisory Board. Inductive/deductive content analysis was used to identify themes. The results identified an overall tension between distrust of researchers and a compelling desire to engage in dementia research. This overarching theme was supported by six themes that provided insights about the multiple layers of distrust, as well as expectations about the appropriate conduct of researchers and academic institutions. Strong commitment to the community was identified as a priority. The findings suggest that a paradigm shift is needed to increase the representation of African Americans in dementia research. In this new paradigm, earning the trust of African American communities becomes a systemic endeavor, with academic, state and national institutions deeply committed to earning the trust of African American communities and guiding researchers in this endeavor. The findings also generated actionable recommendations to help improve representation of African American older adults in dementia research.

